# Viral attenuation by Endonuclease G during yeast gametogenesis: insights into ancestral roles of programmed cell death?

**DOI:** 10.15698/mic2020.02.705

**Published:** 2019-12-17

**Authors:** Jie Gao, Sabrina Chau, Marc D. Meneghini

**Affiliations:** 1 Department of Molecular Genetics, University of Toronto, Toronto, ON, Canada.

**Keywords:** endonuclease G, viral attenuation, mitochondria, yeast, gametogenesis, programmed cell death

## Abstract

Viruses and other genetic parasites are present in virtually all forms of life. This chronic condition has led to diverse host cell adaptations such as CRISPR and RNAi, whose functions attenuate these parasites. It is hypothesized that programmed cell death (PCD) is an additional adaptation whose origins reside in viral defense. A core event of apoptotic PCD is the regulated release of mitochondrial inter-membrane space proteins into the cytosol, following which these apoptogenic proteins bring about the demise of the cell. The most well studied example of this is found in animals, where the release of mitochondrial cytochrome C nucleates the formation of the apoptosome, which then activates caspase mediated cell death. The release of mitochondrial proteins contributes to PCD in diverse organisms lacking the apoptosome, indicating that regulated mitochondrial release predates the evolution of canonical apoptosis. Using the budding yeast *Saccharomyces cerevisiae*, we recently confirmed an early study showing that Nuc1, a homolog of the mitochondrial apoptotic driver protein Endonuclease G, attenuates cytosolic double stranded RNA (dsRNA) viruses, which are endemic to yeast and many other organisms. Viral attenuation by Nuc1 occurs most prominently during meiosis and in association with its developmentally programmed relocation from the mitochondria to the cytosol. Intriguingly, meiotic viral attenuation by Nuc1 occurs within the context of meiotic PCD of the superfluous mother cell that we have also discovered. These findings are discussed here.

Programmed cell death (PCD) is widely found in unicellular microorganisms, both prokaryotic and eukaryotic, and it is increasingly clear that PCD predates multicellularity. The existence of unicellular PCD presents a challenge to the classical paradigm of altruistic cell death that is more easily understood in multicellular contexts where somatic cell suicide contributes to the success of the germline. Although analogous altruistic scenarios (dying for the colony) have been proposed as a benefit of PCD in prokaryotes, the roles of this process remain murky in the case of the budding yeast *Saccharomyces cerevisiae*, the most prominently studied model of cell death in unicellular eukaryotes [1, 2]. Studies of cell death regulation in *S. cerevisiae* (hereafter referred to as yeast) have been advanced almost exclusively through investigations of genes encoding homologs of known human apoptogenic factors [1]. The purported apoptogenic function of these genes has been largely characterized by analyzing their roles in the demise of yeast subjected to environmental insult. It is debatable whether such artificially induced cell death is representative of intrinsically induced PCD, nonetheless, and they are termed “regulated cell death” (RCD). Recent studies have identified meiotic development as a context of *bona fide* PCD in yeast. Interestingly, the conserved mitochondrial nuclease Endonuclease G (EndoG), an apoptogenic protein in humans, contributes both to yeast RCD and to yeast meiotic PCD. This review focuses on an additional role of EndoG in viral attenuation that accompanies yeast meiotic PCD.

Budding yeast belong to the ascomycota phylum of fungi. The distinguishing feature of ascomycetes is the production of “asci”, or sacs, during sporulation. Spore progeny develop within these sacs, which are the remnant carcasses of the mother cells. Sporulation is induced in response to nitrogen starvation and the presence of a non-fermentable carbon source. In response to these nutritional cues, diploid yeast cells initiate a developmental program, coupling the meiotic production of haploid chromosome complements with their cellularization and differentiation into spores [3]. We have discovered that developmentally PCD of the remnant mother cell occurs as an intrinsic feature of sporulation and is executed by meiotically programmed vacuolar rupture, leading to mother cell autolysis through massive autophagic breakdown of its cellular content [4, 5]. This “vacuplosion” phenomenon appears similar to occurrences of PCD via a process termed mega-autophagy in plants [6]. We refer to this phenomenon in yeast as meiotic PCD.

When sporulating cells encounter an environment with suboptimal carbon levels, half of the meiotic products fail to cellularize [4, 7]. Uncellularized meiotic nuclei are swept up in meiotic PCD and their DNA is fragmented into nucleosome-sized ladders, a prominent feature of mammalian apoptosis [4]. This genomic fragmentation requires *NUC1*, the yeast homolog of the conserved EndoG family of mitochondrial DNA/RNA nucleases, which has also been implicated in stress-induced RCD of haploid cells [4, 8]. Furthermore, mitochondria retained by the suicidal mother cell exhibit a loss of membrane potential (Δψ), a characteristic of many forms of PCD [5]. This observation is consistent with the hypothesis that mitochondrially-released Nuc1 during yeast meiotic PCD accomplishes nucleosomal laddering, paralleling the role of mitochondrial membrane permeabilization in the release of apoptogenic proteins during mammalian PCD [9]. We recently confirmed that Nuc1 accumulates in the cytosol of meiotic cells in a developmentally programmed manner, supporting this model [10].

Despite its role in genome fragmentation, the functional significance of Nuc1 has remained obscure, as loss of *NUC1* is inconsequential for the execution vacuolar rupture, the executioner of meiotic PCD [4]. This is reminiscent of mammalian apoptosis, where EndoG promotes DNA fragmentation but is nonessential for PCD [11, 12]. A narrow interpretation of these findings is that DNA fragmentation by Nuc1/EndoG serves only a superfluous “clean up” function during PCD. However, the pervasive association of mitochondrially driven genome fragmentation pathways with PCD in eukaryotes suggests some alternative and ancient function underlying their association. A better understanding of these pathways may provide insights into the paradoxical evolution of the cell's ability to kill itself and illuminate additional targets of extra-mitochondrial Nuc1/EndoG.

Our recent study presents evidence that highlights viruses as targets of Nuc1 during meiotic PCD [10]. Indeed, the host-virus arms race is a ubiquitous feature of life and has been proposed to underlie the evolution of PCD [13]. Yeast are chronically infected with cytosolic double stranded RNA (dsRNA) viruses that have no extracellular phase and are exclusively transmitted through the cytoplasm [14]. The most prominently studied of these comprise the “Killer” system, which involves a paired system of dsRNA viruses called L-A and M.

The double-stranded L-A genome encodes for virion particles with RNA-dependent RNA polymerase activity, which carry out L-A replication and transcription. Holes in the virion permit the extrusion of single-stranded RNA transcripts into the cytoplasm to encode for proteins that comprise the virion. Encapsidation of the viral transcripts within nascent virion particles completes the lifecycle of these intracellular parasites [14]. The M genome is housed and propagated by the L-A virion through the same mechanisms as described for L-A; M is thus completely dependent on and is, in fact, a parasite of L-A [14]. M only encodes for a secreted toxin that kills neighboring cells lacking the M genome, which confers immunity. One explanation for why yeast tolerate L-A is that it is required for maintenance of the M virus, which may provide ecological benefits as a weapon against other yeasts [15]. Nevertheless, the host cell encodes an antiviral system to keep these viruses in check: the *SKI2/3*, and *8* (superkiller) genes encode subunits of the cytosolic Ski complex (SkiC), which prevents Killer overproduction in vegetative cells [14].

Taking cues from a study documenting an L-A attenuation defect in haploid *nuc1*Δ mutants, we investigated meiotic viral attenuation as a candidate function for cytosolic Nuc1 [16]. We found that *NUC1* is essential for viral attenuation of M in parallel to SkiC during sporulation. Mutant spores lacking both *NUC1* and *SKI3* exhibited lethality due to massive intracellular accumulation of Killer toxin [10]. Killer toxin is secreted via the secretory pathway in vegetative cells, and the secretory pathway is re-routed inwards to accomplish pro-spore membrane biogenesis during sporulation [3]. These secretory pathway alterations seem likely to make Killer attenuation essential for spore development. The Killer-dependent lethality of *nuc1*Δ *ski3*Δ mutants allowed us to use developmental genetic approaches to identify maternally acting *NUC1* as crucial for viral attenuation. This maternal period corresponds with when cytosolic Nuc1 was most robustly detected. Further, the yeast VDAC (voltage-dependent anion channel)-encoding genes *POR1/2* were found to act in a *NUC1* antiviral pathway and *POR1* was required for cytosolic accumulation of Nuc1 in the meiotic mother cell. Thus, we posited that VDACs facilitate meiotically programmed relocation of Nuc1 to the cytosol. This population of Nuc1 consequently contributes to Killer attenuation, perhaps by targeting M (and/or L-A) RNA for digestion [10].

The above model presents a tidy explanation for our findings but needs further study. Open questions remain concerning the mechanistic basis and developmental regulation of Nuc1's dual roles in viral attenuation and meiotic PCD. Although Nuc1 enzymatic activity is required for attenuation, there is only circumstantial evidence for direct attenuation of Killer by cytosolic Nuc1 [10]. Indeed, Liu and Dieckmann identified L-A attenuation by Nuc1 in vegetative haploid cells, whereas we did not detect Nuc1 in the cytosol of vegetative cells [16]. The possibility that cytosolic Nuc1 does not accomplish viral attenuation and instead solely facilitates genomic DNA fragmentation cannot be discounted. In such a scenario, *NUC1* may function within mitochondria to affect viral attenuation. This alternative model does not, however, offer a parsimonious explanation for earlier findings demonstrating a similar L-A attenuation role for *POR1* in vegetative haploid cells and our *POR1/2* findings in sporulating cells [10, 17].

The evidence available supports the hypothesis that cytosolic Nuc1 does facilitate viral attenuation in vegetative cells, where protein levels below our detection threshold could be sufficient to accomplish attenuation. In this scenario, a capacity for leaky VDAC-mediated Nuc1 relocation may have been harnessed and accentuated during meiosis, where viral attenuation appears to be more crucial and/or when cytosolic Nuc1 is needed to accomplish other roles. For example, cytosolic Nuc1 may participate in removal of age-accumulated rDNA circles, contributing to sporulation-mediated lifespan rejuvenation [18]. A more complete cell biological understanding of the viral life cycles will be necessary to address these models, as will studies of the Nuc1/virus (and Nuc1/rDNA circle) interaction.

It is now a doctrine of the apoptosis field that apoptogenic proteins access the cytosol via their regulated release from mitochondria. Decades ago, several key *in vitro* and cell-free studies provided support for this model [19–21], leading to the widespread interpretation of *in vivo* observations of mitochondrial proteins in the cytosol as *prima facie* evidence of release. We have similarly advanced the observation of Por1-dependent Nuc1 cytosolic accumulation as evidence of Nuc1 mitochondrial release [10]. An alternative hypothesis explaining our findings, however, is that Por1/2 mediates the cytosolic accumulation of newly synthesized Nuc1 by interfering with its mitochondrial import. Consistent with this hypothesis, we observed the mobility of cytosolic Nuc1 to be slightly retarded compared with mitochondrial Nuc1 [10]. As many proteins undergo cleavage of N-terminal sequences following their mitochondrial import, this slightly larger form of Nuc1 in the cytosol may indicate that it was never imported, and thus could not have been released.

The alternative targeting model posits that Nuc1, which appears to contain a bipartite presequence, accumulates in the cytosol of sporulating cells through changes in Nuc1 trafficking as opposed to release of mitochondrially localized Nuc1. Localization of nuclear-encoded mitochondrial intermembrane space proteins is directed by two categories of targeting sequences: twin cysteine motifs and bipartite presequences [22]. Both categories of proteins cross the mitochondrial outer membrane via the translocase of the outer membrane (TOM) complex, which can shift between a trimeric and dimeric state depending on the presence or absence of Tom22, respectively [23]. Por1 has been shown to regulate the equilibrium between the trimeric and dimeric complexes via physical interaction with Tom22, acting as a “sink” to sequester Tom22 away from the core TOM complex [23]. Shifts in the equilibrium between the dimeric and trimeric forms of the TOM complex affect import of the two classes of mitochondrial proteins: the dimeric complex favours the import of twin cysteine motif proteins and the trimeric complex facilitates the import of bipartite sequence proteins [23].

In the alternative targeting model, Por1/2 promote the formation of the dimeric TOM complex in sporulating cells by sequestering Tom22, perhaps through meiotic induction of Por2, which we showed occurs concurrent to Nuc1 cytosolic accumulation [10]. An equilibrium shift towards the dimeric form of the TOM complex may prevent efficient mitochondrial import of Nuc1, leading to cytosolic accumulation of the protein. This model explains the defects in cytosolic Nuc1 accumulation and viral attenuation we observed in sporulating VDAC mutants, as the trimeric TOM complex would be favoured in these mutants, resulting in efficient mitochondrial import of Nuc1. Therefore, additional studies are needed to determine the mechanism of Nuc1 relocation and examine its conservation with mitochondrial protein release in mammalian apoptosis.

The ongoing investigations into yeast meiotic PCD may yield insight into the evolutionary origins of apoptosis. Evidence for the adaptive role of conserved PCD pathways in a “selfish” unicellular organism, in the form of common effectors of cell death and antiviral defense during yeast meiotic PCD, supports the model wherein cell death mechanisms were co-opted from pro-survival pathways [2]. The dual roles of yeast Nuc1/EndoG in PCD and antiviral defense recall the apoptosis-immunity connection widely observed in prokaryotes and mammals. It seems likely that there are additional conserved elements of mammalian apoptosis and meiotic PCD that have yet to be discovered. We anticipate that a deeper understanding of yeast meiotic PCD and other forms of unicellular RCD will shed further light on how cellular suicide evolved.
